# Drug-Resistant Tuberculosis in High-Risk Groups, Zimbabwe

**DOI:** 10.3201/eid2001.130732

**Published:** 2014-01

**Authors:** John Z. Metcalfe, Salome Makumbirofa, Beauty Makamure, Charles Sandy, Wilbert Bara, Stanley Mungofa, Philip C. Hopewell, Peter Mason

**Affiliations:** Curry International Tuberculosis Center–San Francisco General Hospital, San Francisco, California, USA (J.Z. Metcalfe, P.C. Hopewell);; Biomedical Research and Training Institute, Harare, Zimbabwe (S. Makumbirofa, B. Makamure, P. Mason);; National Tuberculosis Control Program, Harare, Zimbabwe (C. Sandy);; Harare City Health Department, Harare, Zimbabwe (W. Bara, S. Mungofa);; University of Zimbabwe College of Health Sciences, Harare (P. Mason)

**Keywords:** drug-resistant tuberculosis, HIV/TB, retreatment tuberculosis, Zimbabwe, mycobacteria, tuberculosis, Mycobacterium tuberculosis, antimicrobial resistance, bacteria, MDR TB

## Abstract

To estimate prevalence of multidrug-resistant tuberculosis (MDR TB) in Harare, Zimbabwe, in 2012, we performed microbiologic testing on acid-fast bacilli smear-positive sputum samples from patients previously treated for TB. Twenty (24%) of 84 specimens were consistent with MDR TB. A national drug-resistance survey is needed to determine MDR TB prevalence in Zimbabwe.

Emergence of multidrug-resistant tuberculosis (MDR TB) in sub-Saharan Africa poses a major risk to further destabilization of regional TB control programs. Yet, fewer than half of the 46 countries in the World Health Organization (WHO) African Region have provided representative drug-resistance data, and only 10 have reported data since 2007 (*1*).

Zimbabwe has among the highest estimated TB incidence per capita (603/100,000 population) in the world (*2*). Sixteen percent of adults are HIV infected, and approximately three-quarters of active TB cases occur among persons with HIV (*3*). Increasing prevalence (*4*) and epidemic spread (*5*) of MDR TB in neighboring southern Africa countries, sociopolitical instability with economic collapse and severe health system disruptions in 2007–2008 (*6*), and anecdotal reports of MDR TB among Zimbabwean returnees (*7*) may herald an increase in MDR TB prevalence within the country. Formal national surveillance for drug-resistant TB has not been undertaken in Zimbabwe since 1995. To improve early detection and estimate the prevalence of MDR TB, we performed extensive microbiologic testing on samples from consecutive patients with presumptive drug-resistant TB in Harare, Zimbabwe.

## The Study

From November 15, 2011, to November 15, 2012, we prospectively recruited persons suspected of having drug-resistant pulmonary TB within Harare (metropolitan population 2.8 million, 2009), Zimbabwe. Those with suspected drug-resistant TB were defined as persons with cough, fever, night sweats, or weight loss and either 1) a history of >1 month of prior treatment (relapse, treatment after default, or treatment failure) (*8*), or 2) contact with a person with known or suspected drug-resistant TB. Because early identification of drug-resistant TB was an objective of the study, all persons who had smear-positive sputum samples between month 3 and month 5 of anti-TB treatment (“late smear conversion”) were also enrolled. Subjects were recruited from outpatient clinics within Harare’s 2 infectious diseases referral hospitals, health clinics in southern high-density suburbs, and Epworth (a locality outside Harare). For logistical reasons, not all polyclinics in Harare could be included. Compared with the number of notified sputum smear–positive retreatment patients in Harare in 2011 (the most recent year for which data are available), ≈60% of the population base was likely included in our sample.

Ethical approval was obtained from the Medical Research Council of Zimbabwe, the Institutional Review Board of the Biomedical Research and Training Institute, and Human Research Protection Program, University of California, San Francisco. Laboratory methods were undertaken as reported (*9*). In brief, 2 solid (Löwenstein-Jensen [LJ] media) and 2 liquid (BBL MGIT Mycobacterial Growth Indicator Tubes; Becton Dickinson, Sparks, MD, USA) cultures for mycobacteria were performed for each patient in an external quality-assured laboratory. Ziehl-Neelsen staining was used to confirm growth of mycobacteria in all test-positive tubes. Indirect drug susceptibility testing (DST) was performed on all *M. tuberculosis* isolates by using the absolute concentration method on LJ media. In addition, culture for mycobacteria and direct DST were also performed by using the microscopic-observation drug-susceptibility (MODS) assay (TB MODS Kit, Hardy Diagnostics, Santa Maria, CA, USA) in accordance with published procedures.

Of 240 recruited patients, 25 (10%) were from outside Harare provincial limits, and 2 had microbiologically confirmed MDR TB before enrollment; both patients had been referred from South Africa. Of the remaining 213 patients (Table), 203 (95%) had history of prior TB treatment, and 10 (5%) were new patients with known contact with a person who had suspected MDR TB. Of 211 patients, 157 (74%) were HIV infected. Most patients (207/213; 97%) were enrolled at an outpatient setting; 40 (19%) had previously been hospitalized. Twenty-six (35%) of 75 patients had traveled outside of Zimbabwe in the previous 2 years. Neither prior hospitalization (p = 0.51) nor travel outside Zimbabwe (p = 0.94) was associated with MDR TB.

**Table:** Characteristics of participants in study of MDR TB, Harare, Zimbabwe, 2011–2012*

Characteristic	MDR-TB, n = 25	Monoresistant TB, n = 14	Drug-sensitive TB, n = 90	Unconfirmed TB, n = 84	p value
Age, median (IQR)	34 (27–42)	35 (29–45)	37 (30–44)	39 (32–48)	0.67
Male, no. (%)	13 (52)	6 (43)	58 (64)	48 (57)	0.36
Retreatment category, no. (%)					
Treatment failure	9 (36)	0	9 (10)	21 (25)	<0.001
Late smear conversion	8 (32)	1 (7)	30 (33)	13 (16)	
Default	0 (0.0)	2 (14)	13 (14)	7 (8)	
Relapse	7 (28)	7 (50)	34 (38)	34 (41)	
New	1 (4)	4 (29)	1 (1)	4 (5)	
Sputum smear positivity, no. (%)	20 (80)	7 (50)	52 (53)	6 (7)	<0.001
HIV infection, no. (%)	18 (72)	13 (93)	57 (65)	69 (82)	0.02
Antiretroviral treatment, no. (%)	14 (78)	10 (77)	41 (72)	52 (75)	0.95
Time receiving TB treatment, median (IQR)	98 (4–175)	0 (0–3)	31 (2–103)	101 (5–186)	0.03
Prior treatment courses, no. (%)					
None	8 (32)	5 (36)	30 (33)	21 (25)	0.52
1	12 (48)	7 (50)	40 (44)	40 (48)	
≥2	5 (20)	2 (14)	20 (22)	22 (27)	

*Retreatment classification was defined according to international standards (*8*); late smear conversion was defined as sputum AFB smear-positivity after month 3 but before month 5 of treatment. Monoresistant TB was defined as resistance to either isoniazid or rifampin, but not both. The denominator for antiretroviral treatment included persons with HIV infection. MDR TB, multidrug-resistant tuberculosis; IQR, interquartile range; AFB, acid-fast bacilli.

Among 84 TB case-patients with positive sputum-smear results, 20 (24%; 95% CI 15%–34%) had MDR TB. When patients with both smear-positive and smear-negative results were considered, 25 (12%; 95% CI 8%–17%) had MDR TB, 14 (7%; 95% CI 4%–11%) had monoresistant TB, and 90 (42%; 95% CI 36%–49%) had drug-sensitive TB. Three (12%) of 25 MDR TB patients reported prior TB treatment in South Africa. Among 84 patients (39%; 95% CI 33%–46%), *M. tuberculosis* could not be confirmed by culture on LJ media, manual MGIT, or MODS. Among 84 patients with unconfirmed TB, 21 (25%) had a reported sputum smear–positive specimen (any grade) within a public-sector laboratory in the 2 weeks before enrollment.

To our knowledge, this prospective study from the capital and largest city in Zimbabwe provides the first assessment of MDR TB in the country since 1995. Among a representative sample of retreatment patients in Harare, a high proportion of case-patients (24%) had MDR TB. Although these case-patients were not a random sample of the TB population, these data are worrisome, given regional immigration patterns and ongoing challenges within the health system.

Despite a 2009 World Health Assembly resolution to achieve “universal access to diagnosis and treatment of MDR TB and XDR TB [extensively drug-resistant TB]” by 2015, the extent and course of the MDR TB epidemic in the WHO African Region outside of South Africa (with the largest absolute number of MDR TB patients in the continent) remain poorly described. Although MDR TB prevalence has likely remained stable in Zambia, Malawi (*10*), and Mozambique (*11*), it has increased in Botswana (*4*) and Swaziland (*12*). The current WHO estimate of MDR TB prevalence among retreatment patients in Zimbabwe (8.3%; 95% CI 3%–22%) (*2*) is based on a 1995 subnational drug-resistance survey in which 3 of 36 sputum smear–positive patients had MDR TB. According to this estimate, 970 (95% CI 406–1,980) MDR TB cases occur annually, although only ≈10% are currently detected (*13*). In our sample, one-quarter of smear-positive, previously treated patients had MDR TB, suggesting that MDR TB cases in the country may exceed 1,000 among retreatment patients alone. Although drug-resistance surveys typically sample only those with sputum smear–positive results, we also enrolled retreatment patients with presumptive smear-negative results, given the known poor sensitivity of smear microscopy among HIV-infected patients in sub-Saharan Africa and the likelihood of underrepresentation of HIV-infected persons (*14*). Although fluoroquinolone resistance has been detected (A. Jindani, principal investigator of RIFAQUIN trial, pers. comm.), lack of further second-line DST has thus far precluded identification of extensively drug-resistant TB in the country.

Our report has limitations, however. Although a substantial proportion of nationally notified TB cases (≈15%) occur in Harare, regional variation in incidence and prevalence of MDR TB is known, in particular along Zimbabwe’s border. Treatment with first-line drugs before microbiological investigation differentially selects for drug-resistant organisms. Most patients had been previously treated, and drug resistance within this group reflects a combination of acquisition, reinfection, and primary infection with a drug-resistant strain and subsequent treatment failure. A valid estimate of MDR TB prevalence among new patients without risk factors for drug resistance is not currently available, although this group likely accounts for most MDR TB cases in the WHO African Region and globally (*1*). Finally, lack of genotyping data precludes identification of unsuspected transmission patterns, including transnational spread.

## Conclusions

A representative drug-resistance surveillance study is urgently needed to estimate the prevalence of MDR TB in the general population of Zimbabwe. A comprehensive response is needed, including an increase in quality-assured laboratory capacity for culture and DST (including second-line drugs), reassessment of infection control practices, and expanded investigation of contacts with drug-resistant TB. Finally, overall strengthening of directly observed therapy, short course (DOTS), improvements in health service delivery, and political and socioeconomic stabilization are essential to prevent expansion of MDR TB in Zimbabwe.
